# Sex specific effects of irisin on the skeleton

**DOI:** 10.1038/s41413-026-00542-4

**Published:** 2026-06-10

**Authors:** Anika Shimonty, Lynda F. Bonewald

**Affiliations:** 1 Brigham and Women’s Hospital, Harvard Medical School, Boston, MA USA; 2 Indiana Center for Musculoskeletal Health, Department of Anatomy, Cell Biology and Physiology, Department of Orthopaedic Surgery, Indiana University School of Medicine, Indianapolis, IN USA

**Keywords:** Bone, Endocrinology

## Abstract

Irisin, the circulating protein derived from cleavage of fibronectin type III domain containing protein 5 (FNDC5) has attracted considerable attention regarding its positive effects on a number of organs/tissues such as fat, brain, and heart. However, effects on bone can appear to be either positive or negative depending on context and sex. Sex differences have also been described in Alzheimer’s disease also emphasizing sex differences in response to irisin. Here in this review, we outline and focus on the effects of irisin on osteoblasts, osteoclasts, and osteocytes, murine models, and human studies. The consideration of sex effects is necessary especially in the context of irisin serving as a therapeutic.

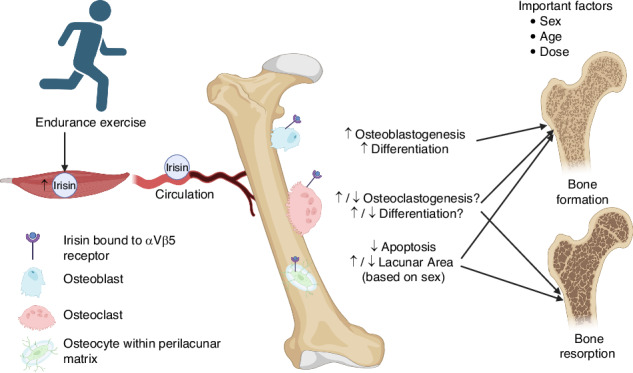

## Introduction

Skeletal muscle cells produce and secrete a variety of metabolites, peptides, and cytokines collectively known as myokines. Irisin is one such myokine discovered in 2012.^1^ Named after the mythical messenger of the Greek gods, Iris, irisin appears to function as a myokine by carrying “messages” from skeletal muscle to other cells and organs. A plethora of studies have been carried out in recent years to determine the exact nature of these messages, which organs and tissues are being targeted, the molecular mechanisms responsible, and their potential clinical relevance. A recent literature review has shown 1 510 articles and reviews in the field of irisin from 2012 to 2021,^2^ which is increasing annually. In September of 2025, a PubMed search of “irisin” resulted in 2 562 articles and reviews, showcasing the exponential increase in research in the field.

It began in 2002, when two groups independently discovered a gene expressed in multiple tissues including skeletal muscle with a sequence placing it in the fibronectin type III superfamily.^3,4^ Later this gene was named fibronectin type III domain containing protein 5 (FNDC5). FNDC5 first came into the limelight when Bostrom et al. showed that increased expression of the PPARγ coactivator-1 α, *PGC1α*, in skeletal muscle increased the expression of FNDC5 and showed that the protein product is then cleaved by an unknown protease to release the secreted hormone, irisin.^1^ Exercise, primarily resistance exercise, induces an increase in PGC-1α, concomitant with an increase in FNDC5 and its subsequent cleavage to generate irisin. The cleavage process and responsible enzyme are yet to be discovered. The ADAM family of disintegrins and matrix metalloproteinases has been proposed to cleave FNDC5 into irisin in C2C12 myotubes.^5^ The cleaved form of FNDC5, irisin, can reach the circulation and travel to distant organs, thus acting in a paracrine manner, especially in bone. It has recently been shown that extracellular vesicles, EVs, isolated from blood contain irisin.^6,7^ It is not clear how a membrane protein can be cleaved, releasing a segment of the external portion of this membrane protein and then become incorporated into an EV. Also, something else that is not clear is whether the short half-life of injected irisin^8^ of 1 h can be extended in the form of EVs. This is a new area of investigation.

Irisin induces an increase in uncoupling protein 1 (UCP1) in white adipose tissue, which in turn induces browning, thereby meriting the classification as a myokine. Other tissues that are targeted by irisin include brain, bone, cardiomyocytes, hepatocytes, and others.^1,9–17^ Even though FNDC5 gene expression is detected in heart, adipose tissue, kidney, testes, brain, peripheral nerves, pancreas, spleen, stomach, and other tissues as well, skeletal muscle is the primary producer.^18–20^ Our group have found little or no expression of *Fndc5* mRNA in primary osteoblasts and primary osteocytes (transcriptome analysis with a raw count of 8–12), however both skeletal muscle (gastrocnemius) and C2C12 myotubes have high expression of *Fndc5* (transcriptome raw count of 512–1 000).^21^ Therefore, irisin does not appear to be made by mature osteoblasts and osteocytes.

The major receptor for irisin is the alpha V beta 5, αVβ5, an integrin originally identified and characterized from MLO-Y4 osteocyte-like cells.^16^ Irisin stimulated the “integrin-like” signaling that includes pFAK, pZxyn and pCREB in both osteocytes and fat. Integrin inhibitors such as echistatin and RGD peptides prevented irisin-induced signaling. Several studies have validated αVβ5 as the major receptor for irisin in many other cell types and its signaling pathways have been expanded to also include MAPK, ERK, c-jun, p38, and ERK5 (Please see review by Hu et al.^22^). The activation of numerous pathways by the αVβ5 integrin suggests cell-specific interactions with other surface and intracellular modulators of integrin signaling.

## Paracrine effects of irisin on the brain, heart, and liver

Exercise has a highly beneficial effect on brain health and function, and irisin has been proposed to be responsible for conferring these positive effects.^23^ Wrann et al. have shown that FNDC5 gene expression increased in the hippocampus in mice after endurance exercise. This in turn increased neuroprotective gene expression in the hippocampus, including brain-derived neurotrophic factor (*Bdnf*).^9^ In a recent study, the same group has shown that irisin plays an important positive role in cognitive functions in exercise, aging, and degenerative diseases such as Alzheimer’s disease (AD). Using the tail-vein injection method to deliver exogenous irisin, they have also shown that irisin can cross the blood-brain barrier.^12^ A different group has demonstrated a decreased level of FNDC5/irisin in the AD hippocampus and cerebrospinal fluid in both humans as well as murine models of AD. Inhibiting FNDC5/irisin blocked the protective effect of exercise in AD mice, and overexpression of FNDC5/irisin improved memory.^24^ Irisin-treated primary astrocyte cell conditioned media has protective effects against β-amyloid-induced toxicity in in vitro neuronal cell cultures.^25^ Irisin has also been shown to be protective against oxygen-glucose deprivation-induced neuronal injury in in vitro PC12 neuronal cell culture.^26^

Cardiomyocytes have been shown to both produce and be regulated by irisin. In a mouse myocardial infarction model, irisin has been shown to increase cardiac regeneration and improve function.^27^ A separate study has shown a therapeutic effect of intraperitoneally injected recombinant irisin (r-irisin) in adult male mice with myocardial infarction due to increased angiogenesis.^28^ Irisin has also been shown to reduce cardiac hypertrophy and fibrosis due to pressure overload^5^ and reduce dysfunction in sepsis-induced cardiomyopathy.^29^

Irisin has been shown to be protective against liver steatosis by decreasing oxidative stress and inflammation.^30^ In vitro studies using AML12 cells and mouse primary hepatocytes showed a decrease in lipid accumulation and oxidative stress upon treatment with r-irisin.^31^ Young obese male mice intravenously injected with a lentivirus containing the FNDC5 gene have increased energy expenditure, insulin resistance, and lipolysis.^32^ Fasted FNDC5 null mice have increased steatosis and lipogenesis, which could be rescued using rapamycin, an inhibitor for mammalian target of rapamycin (mTOR) complex 1.^33^ This suggests a potential mechanism of action for FNDC5 in the liver via the AMPK/mTOR pathway. In a mouse model of high-fat diet-induced non-alcoholic fatty liver disease (NAFLD), treadmill exercise decreased hepatic fibrosis and steatosis, proposed to potentially be through increased levels of irisin.^34^ Interestingly, in a human study with NAFLD patients, circulating irisin levels were higher in patients with higher fibrosis^35^ potentially as a compensatory mechanism against the fibrosis. Therefore, irisin is generally is thought to have beneficial paracrine effects on liver, heart, and brain.

## Irisin and bone

Bone is a dynamic organ that interacts with muscle in both mechanical and biochemical ways.^36,37^ The three bone cell types: osteoblasts, osteoclasts, and osteocytes work in tandem to maintain bone health and integrity. Osteoblasts are bone-forming cells that function in bone matrix protein secretion through prodigious production of collagen type 1, followed by mineralization, whereas osteoclasts are bone-resorbing cells that function to remove bone through the simultaneous production of proteases that degrade bone proteins and the release of protons into their resorption lacunae to dissolve mineral.^38,39^ Osteocytes are the cells embedded in the bone matrix and descend from osteoblasts to function as long-lived master regulators of both bone formation and bone resorption, especially in response to mechanical loading and hormonal regulation.^36^ Factors produced by loaded osteocytes that produce positive effects include PGE_2_ and wnts^37,40,41^ while factors produced by unloaded osteocytes produce negative effects, such as fibroblast growth factor, FGF9^42,43^ and receptor activator of nuclear factor kappa-Β Ligand, RANKL.^37,44^ Different myokines and muscle metabolites including brain derived neurotropic factor, BDNF, β-aminoisobutyric acid (BAIBA), myostatin, and interleukin-6 (IL-6) can directly regulate bone functions by interacting with these bone cells.^45–47^ Studies support the concept that there is crosstalk and communication between bone and muscle.^45,46,48^ It has been proposed that the beneficial effects of exercise are mediated through both myokines and osteokines,^45,46,49–51^ however many new publications are assuming that it is mainly irisin that is responsible for the positive effects of exercise. It is highly likely that other myokines and potentially osteokines may amplify the effects of irisin, either additively or synergistically.

Various studies have examined the effects of muscle factors on bone cells; however, some studies investigating irisin effects on bone appear to be contradictory, unlike the effects of irisin on other organs. In this review, we provide potential explanations for these discrepancies.

## Mouse models are used to study the effects of irisin on bone

To date, several groups have generated mouse models with global deletion of FNDC5. One group generated an irisin knockout mice by using Transcription activator-like effector nuclease (TALEN) technology to clip the exon 3 18th and 19th nucleotide, which created a frameshift mutation resulting in irisin deletion. They found that at 24 weeks of age, the KO mice had decreased tibial bone strength and femoral bone mass. These mice also had higher RANKL and tartrate-resistant acid phosphatase (TRAP) levels, suggesting increased osteoclastic bone resorption, however, the serum levels of alkaline phosphatase (ALP) and osteocalcin (OCN) were not different from the wild-type, WT controls.^52^ These results suggest that irisin is potentially targeting the osteoclast and not necessarily the osteoblast. Unfortunately, information as to whether males or females were used to generate this data was never provided.

Another, different germline-deleted FNDC5 global knockout mouse model was generated by crossing EIIa-cre mice with FNDC5 floxed mice with deletion of exons 2 and 3. These mice have no FNDC5, and subsequently no irisin. Interestingly, these mice, male or female, showed no differences in cortical bone architecture under normal conditions compared to WT, except for lower RANKL expression.^16^ These results suggest that the deletion of irisin has no effect on bone development, in contrast to the TALEN-generated model described above.

Estell et al. generated a mouse model with muscle-specific overexpression of FNDC5/irisin using an *Mck* (Muscle creatinine kinase) promoter.^53^ The female mice had significantly lower trabecular bone volume fraction at 2 and 4.5 months of age; however, no significant difference was observed at 13 months of age despite similar trends. (Of note, female mice lose trabecular bone at 5 months and upwards, which makes quantification and statistical analysis more difficult in older female mice^47^). In vitro, supporting in vivo observations in female mice, irisin was found to directly stimulate the differentiation of osteoclast progenitors and promote bone resorption.

Zhu et al. have generated a mouse model with the intention to delete irisin in osteoblasts by crossing an Osterix-Cre with a floxed FNDC5 to determine if irisin has a function in osteoblasts. These mice show impaired bone development and decreased bone density due to both an increase in osteoclastic genes and a decrease in osteoblastic genes.^54^ One would interpret these studies to mean that irisin is produced by osteoblasts and has a positive autocrine effect on bone formation and that irisin secreted by muscle and other tissues is not responsible for targeting bone. However, we have reported low to no expression of FNDC5 in both primary osteoblasts and osteocytes, in contrast to high levels found in murine gastrocnemius and C2C12 cells.^21^ One explanation for the bone phenotype in these mice is that osterix has also been shown to be expressed in other tissues than bone or osteoblasts.^55–57^ Another explanation is that the Cre could be targeting a progenitor of osteoblasts. These potential caveats should be taken into consideration when assessing the function of irisin in bone.

We have utilized the germline FNDC5 deletion model referred in ref. ^16^ to study the effects of irisin on osteocytes.^21^ We found no expression of FNDC5 gene in either osteocytes or osteoblasts. There was no difference between the WT and FNDC5-null female mice skeleton structure or strength at 4-5 months of age. Osteocyte transcriptomes and lacunar area were relatively similar in WT and null female mice. However, the null female mice had fewer TRAP-positive osteocytes compared to age-matched WTs. On the other hand, at 4–5-months, male FNDC5-null mice had more yet weaker bone compared to WTs. Null mice also had fewer TRAP-positive osteocytes but no significant difference in osteocyte lacunar area. We also found significant differences between the WT and null male mice osteocyte transcriptomes, especially in fatty acid and lipid transport and metabolism pathways.^21^ However, when challenged with calcium deficiency, the null females were protected from bone loss, while the null males were not. In summary, FNDC5 global deletion had few effects on the female skeleton and minor effects on the male skeleton under normal conditions, but significant differences with the challenging condition of calcium deficiency.

In summary, based on the phenotypes of transgenic FNDC5 mouse models, the effects of irisin on bone are unclear.

## Effect of recombinant irisin (r-irisin) on osteoblasts and osteoclasts

### Osteoblasts

Colaianni et al. performed a study where they collected and cultured myoblasts from 2-month-old male mice allowed access to wheel running to test whether myoblast-secreted factors would have an effect on osteoblast differentiation. Conditioned media from myoblasts from wheel- running mice induced higher expression of alkaline phosphatase (*Alp*) and collagen I (*Col I*) in bone marrow stromal cells than myoblasts from non-exercised controls and this effect was blocked by an irisin-neutralizing antibody. From these experiments, the authors concluded that the increased osteoblastogenesis is irisin-dependent.^58^ The same group showed that r-irisin counteracted the negative effects of microgravity by maintaining the expression levels of Runt-related transcription factor 2 (*Runx2*), Activating transcription factor 4 (*Atf4*), Osterix (*Osx*), *Col I*, and Osteoprotegerin (*Opg*) proteins in 3D in vitro osteoblasts from 8-week-old male mice.^59^ R-irisin treatment has also been shown to promote osteoblast proliferation and differentiation in pre-osteoblastic MC3T3 cells and in primary male rat osteoblasts; and promoted osteogenic differentiation in bone marrow stromal cells and bone marrow mesenchymal stem cells.^60–64^

R-irisin injected intraperitoneally exerted a beneficial effect on cortical bones in young male mice by reducing the secretion of osteoblast inhibitors and increasing the activity of osteogenic cells.^65^ Fracture healing was enhanced in young male mice with intraperitoneal injection of r-irisin.^66^ A recent study using 4-week-old male ICR mice with recombinant human bone morphogenic protein 2 (BMP2) implants showed that ectopic bone volume and bone mineral content, BMC, were higher in mice that received r-irisin injection. Gene expression levels of *Runx2*, *Alp*, osteocalcin (*Ocn*), and osteopontin (*Opn*) were increased by recombinant-BMP-2 alone, but significantly enhanced with the addition of r-irisin.^67^ Another group used 8-week-old ovariectomized mice injected with 100 μg/kg r-irisin once a week for 4 weeks and showed a rescue from ovariectomy-induced bone loss in the treated group along with an increase in bone formation rate. They also showed that irisin at 0.1 μg/mL irisin treatment of MC3T3-E1 cells significantly accelerated osteoblast differentiation.^68^

A renal osteodystrophy study used 6–8-week-old male C57BL/6 mice to induce chronic kidney disease (CKD) via nephrectomy, that were subjected to either aerobic exercise or daily r-irisin injection for 4 weeks.^69^ Aerobic exercise increased circulating irisin levels as well as bone formation, mitigating osteodystrophy. R-irisin treatment had similar effects to aerobic exercise. Interestingly, continuous r-irisin injections were necessary to obtain beneficial effects on bones. Discontinuation of treatment after 2 weeks resulted in low BMD in CKD mice. Browning of adipose tissue, an important function of irisin, did not have any effect on the anti-osteodystrophy, as removal of brown adipose tissue did not change irisin treatment outcome. Both aerobic exercise and r-irisin treatment increased cartilage area in CKD mice. Both aerobic exercise and r-irisin increased gene expression levels of osteoblast activation markers *Alpl*, RANKL, adiponectin receptor 1 (*Adipor1*), *Bglap*, *Bmp4*, and osteoprotegerin (*Tnfrsf11b*) but had no effect on the expression levels of osteoclast markers *Ctsk*, *Acp5*, and *Mmp9*. Irisin treatment in combination with a low dose of zoledronic acid and calcitrol had a better outcome compared to irisin alone. None of these treatments generated a renal burden. This group also performed a pilot clinical study with 6 end-stage renal disease patients (both males and females) where 3 of them conducted light aerobic exercise for 12 weeks. Exercise increased circulating irisin levels 1.3-fold and BMD increased after 12 weeks of exercise.^69^ One must consider the small sample size when interpreting these results.

2 and 15-month-old male C57BL/6 mice were administered irisin once a week via the caudal vein for 4 weeks.^70^ Afterwards, bone marrow from these mice was extracted and cultured. Bone marrow stem cells (BMSCs) cultured for osteogenic differentiation with different concentrations of r-irisin showed that high doses of irisin treatment increased both alkaline phosphatase activity and mineralization. Conversely, irisin decreased lipid droplet numbers after adipogenic differentiation of BMSCs. Irisin decreased senescence and enhanced pluripotency of BMSCs derived from aged mice through the wnt/β-catenin pathway. Relative serum irisin levels were lower in aged mice compared to young mice. Irisin treatment in aged mice resulted in significantly higher bone compared to vehicle-treated aged mice. Irisin treatment also partially reshaped the growth plate and osteoid formation in aged mice. Additionally, they observed a decrease in bone marrow adipose tissue formation in aged mice, possibly via the Wnt signaling pathway. This group concluded that irisin modulates BMSC differentiation in aged mice with an increase in osteogenic differentiation and a decrease in adipogenic differentiation through Wnt/β-catenin/ATF4/PPAR-γ signaling.^70^

Hatakeyama et al. conducted a study using 8-week-old male C57BL/6 J mice which were electroporated to introduce HA-tagged N-terminal FNDC5 into the gastrocnemius muscle.^71^ One subset of treated mice also underwent a week of aerobic exercise. However, neither the irisin-treated group nor irisin combined with exercise group had any effect in bone parameters. Osteoblast activity as well as osteocalcin-positive cells were significantly increased compared to control in irisin and exercise combined group, but not in the only irisin-treated group. Osteoclast activity was not affected. Compared to the sedentary control group, *sost* mRNA decreased in both the irisin-treated group and the irisin and exercise combined group. *Rankl*, *Opg*, and *Runx2* levels were unchanged. These results suggest that irisin overexpression positively modulates osteoblast activity only after aerobic exercise.^71^

Fourteen to 16-week-old male Wistar albino rats were used to study the effect of irisin on fracture healing. 0.1 mL of r-irisin injected directly into the fracture site resulted in accelerated healing evident by higher bone volume and strength compared to control, platelet-rich plasma treated, and hyaluronic acid treated groups.^72^ Another recent study using 2-month-old female C57BL/6J ovariectomized mice undergoing intraperitoneal injection of 100 µg/kg r-irisin two times a week for 5 weeks found increased osteoblast activity as well as increased ALP, BGP, and OPG levels compared to ovx-ed control group. Additionally, r-irisin treatment reduced inflammatory cytokines and modulated gut microbiota, suggesting an effect of irisin on bone–gut axis.^73^

**Fig. 1:**
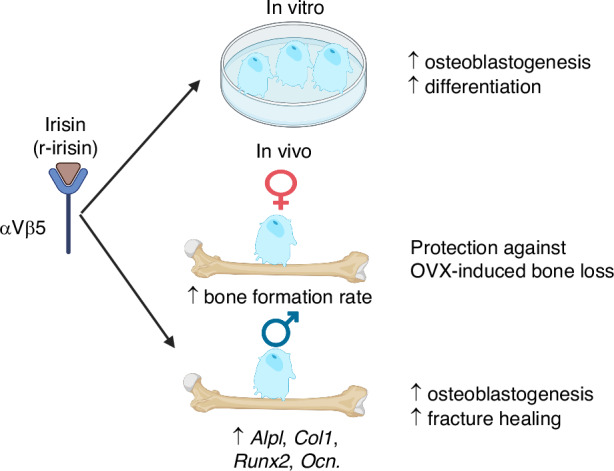
Summary of the proposed effects of irisin on osteoblasts (Created with BioRender.com). αVβ5 is the functional receptor on osteoblasts. Studies utilizing recombinant irisin (r-irisin) have shown an increase in osteoblast proliferation and differentiation in both primary cell cultures and cell lines. Ovariectomized female mice treated with r-irisin were protected against bone loss via an increase in bone formation rate (ref). Male mice had higher fracture healing rate due to higher osteoblast proliferation and function^58–73^. Overall, studies suggest that irisin positively modulates osteoblast proliferation and functions in both females and males

Most of these in vitro and in vivo studies share a consistent finding that irisin promotes osteoblast proliferation and differentiation (summarized in Fig 1).

### Osteoclasts

The effects of irisin on osteoclasts are contradictory. As mentioned above, in an effort to mainly increase irisin production in muscle using muscle-specific overexpression of FNDC5/irisin resulted in female mice with less bone. These authors also showed that in vitro, irisin directly stimulated the differentiation of osteoclast progenitors^53^ and that irisin treatment of osteoclast progenitors resulted in increased mitochondrial content and reactive oxygen species. There was also an increase in maximal oxygen consumption rate and spare capacity. This suggests an increase in mitochondrial respiration and increased osteoclastogenesis.^74^

Two groups have used RAW264.7 cells as a model of osteoclasts. One showed an increase in osteoclast precursor proliferation but a decrease in differentiation in response to r-irisin^75^ while the other also showed an increase in proliferation but also an acceleration of differentiation.^53^ In this study irisin increased proliferation, differentiation, and expression of bone resorption genes including Receptor Activator of Nuclear Factor kappa-Β (*Rank*), Dendrocyte Expressed Seven Transmembrane Protein (*Dcstamp*), Transforming Growth Factor β2 (*Tgfβ2*), ATPase H^+^ Transporting V0 Subunit D2 (*Atp6vod2*), and other markers of osteoclasts.^53^ They have also shown that αVβ5 acts as the irisin receptor in osteoclasts- the same receptor for irisin originally discovered on osteocytes.^53^ Another group using the OCCM-30 cementoblast cell line showed that irisin treatment increased expression of osteoclastogenesis genes including RANKL and IL-6. They hypothesized a positive effect of irisin on cementoblast-mediated osteoclastogenesis.^76^

Several publications suggest that a reduction in circulating irisin may be responsible for both bone and muscle loss as outlined here. In hindlimb unloading/ suspension and sciatic neurectomy murine models, a decrease in circulating irisin in conjunction with both bone and muscle loss was observed.^77^ This decrease of irisin may be due to the reduction in muscle size in these models, as skeletal muscles are the primary producers of irisin. In another study, r-irisin injected intraperitoneally protected against disuse-induced bone and muscle loss in young male mice in the hindlimb suspension model.^78^

A decrease in osteoclastic bone resorption compared to controls was observed in ovariectomized mice and rats with exogenous irisin treatment via intraperitoneal injection.^79,80^

**Fig. 2:**
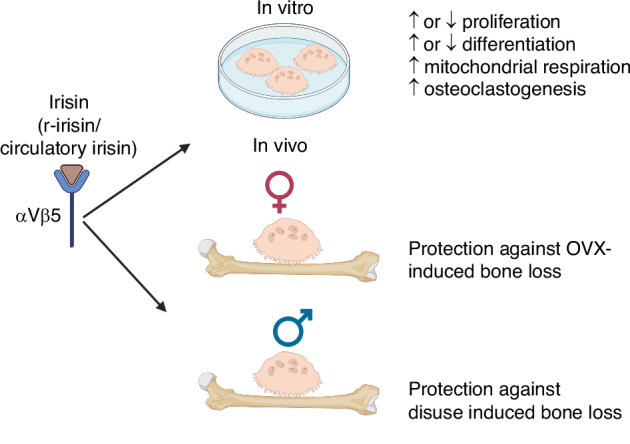
Summary of the proposed effects of irisin on osteoclasts (Created with BioRender.com). In vitro studies using different osteoclast cell lines have resulted in contradictory findings, including either an increase or a decrease in osteoclast proliferation and differentiation. Studies found increased mitochondrial respiration possibly leading to higher osteoclastogenesis. In vivo, treatment with r-irisin protected ovariectomized female mice and rats and hindlimb-unloaded male mice from bone loss via reduction in osteoclast resorption^74–80^. Overexpression of *Fndc5* in female mice resulted in increased osteoclastogenesis^53^. These contradictory findings may be due to dose or duration of treatment and require further studies

The different outcomes in these in vitro studies are not clear but may be attributed to the difference in dose and duration of the treatment, where a shorter treatment promotes osteoclastogenesis and a longer treatment inhibits the process (summarized in Fig 2). The differences in the mouse models could be due to overexpression in one model as compared to the delivery model. Irisin could have different effects depending on tissue and pathological context.

### Effect of irisin on osteocytes

Four of the major functions of osteocytes, the longest living and most abundant bone cell, are: 1) removal and replacement of their perilacunar matrix in response to calcium-demanding conditions, 2) to function as mechanosensors to produce factors that regulate osteoblasts and osteoclasts,^36^ 3) to regulate not only calcium but also phosphate homeostasis,^81,82^ and 4). to function as endocrine cells that target distant organs such as kidney and as addressed here muscle.^37^ In response to mechanical loading, osteocytes release prostaglandin E_2_, PGE_2_ and Wnts, which have beneficial effects on myogenesis and muscle function.^45,83,84^ Unloading of bone causes osteocytes to produce RANKL, the ligand for the RANK receptor, which in turn induces osteoclastic bone resorption,^85,86^, which also has negative effects on muscle mass and function.^87^

Osteocytes are a major participant in bone-muscle crosstalk. Different, distinct factors are produced by osteocytes depending on whether the cells are loaded or unloaded and can reach skeletal muscle tissue through their extensive lacuna-canalicular network and the vasculature system.^37,45,46,83^ In response to unloading, osteocytes produce sclerostin encoded by the gene *Sost*, a negative regulator of bone formation through inhibition of wnt/b-catenin signaling that can also negatively modulate muscle.^43,88,89^ Conversely, mechanical loading decreases *Sost* mRNA levels in osteocytes.^90^ Conditioned media from the MLO-Y4 osteocyte-like cell line has been shown to increase C2C12 myoblast differentiation, which can be inhibited by addition of sclerostin.^91^ Serum sclerostin levels are negatively associated with skeletal muscle mass.^88^ This may be one of the mechanisms whereby disuse or unloading results in both bone and muscle mass loss. Another major player in bone-muscle interactions is the RANK-RANKL-OPG system. Osteoporotic women treated with a RANKL-neutralizing antibody, denosumab, were found to not only have increased bone mass but also have increased appendicular lean mass and handgrip strength.^92^ In ES-2 ovarian cancer tumor-bearing mice, the increase in RANKL was responsible for both muscle functional weakness and atrophy in addition to bone loss, and was reversed with a RANKL-neutralizing antibody treatment.^93^ Therefore, targeting RANKL has positive effects on both bone and muscle.

Osteocytes can also remove and replace their perilacunar matrix under various conditions but especially under calcium-demanding conditions such as lactation. The removal of the bone matrix around the osteocyte lacunae is known as osteocytic osteolysis, but when accompanied by replacement, the process is referred to as perilacunar remodeling.^37^ Osteocytic osteolysis occurs in pathological conditions such as hypophosphatemic rickets, hyperparathyroidism, and cancer.^94^ In contrast, the physiological condition of lactation induces osteocytic osteolysis that is reversed by the replacement of that matrix, called perilacunar/canalicular remodeling.^95–98^ The release of calcium from the bone by osteocytes during lactation is thought to play an important role in providing milk for offspring.^96^ As reviewed later, irisin appears to play a role in this process.

**Fig. 3:**
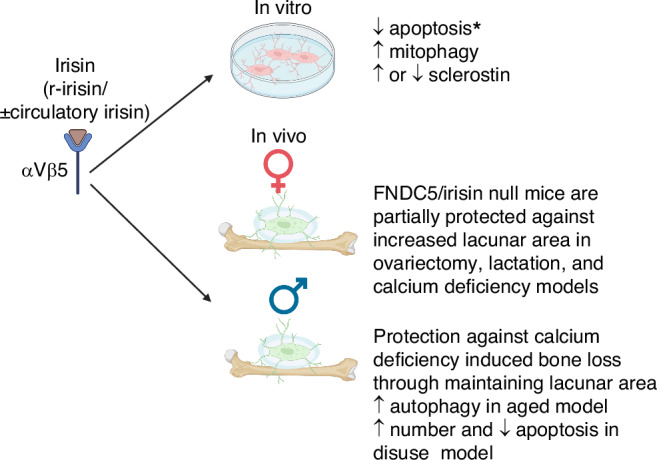
Summary of the proposed effects of irisin on osteocytes (Created with BioRender.com). Multiple in vitro studies have found irisin to have an anti-apoptotic effect on osteocytes (marked * in the figure), possibly due to increased mitophagy. However, sclerostin production has been shown to either increase or decrease in different studies^16^^,^^99–101^. In vivo, FNDC5/irisin-null female mice were protected against bone loss and increased osteocyte lacunar area in multiple bone loss models including ovariectomy, lactation, and calcium deficiency), whereas null male mice bone lose greater amounts of bone compared to wild-type males on a calcium-deficient diet^16^^,^^21^^,^^102^. This points to a sex-specific effect of irisin on osteocytes. Total absence of irisin (in global null mice) and intermittent treatment of r-irisin may have different effects on osteocytes

To date, few studies have been conducted regarding the effects of irisin on osteocytes (summarized in Fig 3). Osteocytes express αVβ5 integrins, shown to be the receptor for irisin.^16^ In the publication by Kim et al., treatment of the MLOY4 osteocyte-like cell line with 1–500 ng/mL r-irisin for 16 h resulted in a decrease in H_2_O_2_-induced apoptosis along with an increase in sclerostin. This suggested that irisin prevents or reduces osteocyte cell death while simultaneously increasing sclerostin to reduce bone formation. In this same study, 9-month-old ovariectomized (ovx) FNDC5 global null mice were protected against ovariectomy-induced trabecular bone loss due to osteocytic osteolysis and osteoclastic bone resorption. Osteocyte lacunar area was significantly smaller in ovx FNDC5 null mice compared to their WT counterparts, indicating reduced osteocytic osteolysis. Interestingly, WT ovx mice had an increased level of circulating irisin compared to WT sham controls.^16^

Similarly, in another study using MLO-Y4 cells treated with 100 ng/mL irisin for 8 or 24 h a decrease in both H_2_O_2_-induced osteocyte apoptosis and in sclerostin with an increase in the pro-survival Bcl2/Bax ratio was observed.^99^ Young hindlimb-suspended male mice treated with r-irisin had higher osteocyte numbers per bone surface compared to vehicle-treated controls due to decreased osteocyte apoptosis. 18-month-old male mice, a male model of age-induced osteoporosis, treated with 100 μg/kg r-irisin once a week for 4 weeks, also showed an increase in the Bcl2/Bax ratio.^99^

He et al. also using the MLO-Y4 cells, have shown that treatment with 100 ng/mL r-irisin for 24 h resulted in higher viability of osteocytes and lower cyclic-stretching-induced apoptosis.^100^ This was accompanied by a decrease in sclerostin and an increase in OPG/RANKL ratio. Young male mice that underwent an anterior cruciate ligament transection followed by r-irisin treatment had improved bone microarchitecture and remodeling compared with the vehicle-treated group. This was thought to be due to increased osteocyte viability, decreased osteocyte apoptosis, as well as decreased TRAP-positive osteoclasts.^100^ All studies to date support that irisin maintains osteocyte viability.

Irisin has been reported to increase autophagy in osteocytes. Li et al. treated 24-month-old male C57BL6J mice with 10 μg/kg/day r-irisin for 8 weeks and found increased autophagy in osteocytes.^101^ The irisin-treated mice had higher BMD, bone volume, and higher physical activity compared to age-matched controls. Treated mice also had both higher numbers of TRAP-positive osteoclasts, higher osteocalcin, and higher sclerostin expression, indicating more robust bone remodeling. In vitro, irisin treatment increased proliferation and mitochondrial activities in MLO-Y4 cells. Mitophagy and Ampk phosphorylation were also increased. The authors surmised that the irisin-induced increase in osteocyte mitophagy helped in the reduction of bone loss with age.^101^

Using the same global FNDC5-null mice as the Kim study,^16^ we examined the effects of irisin on osteocyte functions during periods of calcium deficiency.^21^ We found that adult female null mice were partially protected against lactation and calcium-deficient diet-induced bone loss due to both less osteoclast resorption and less osteocytic osteolysis. This suggests a role for irisin in regulating the release of calcium from bone during calcium-deficient conditions, especially via osteocytes. During lactation, calcium release from maternal bones is important for offspring survival. This indicates a potential role of irisin in modulating successful reproduction. On the other hand, male null mice had more bone under normal conditions; however, they were less resistant to bone loss due to diet-induced calcium deficiency, in part due to the significant increase in osteocytic osteolysis and TRAP-positive osteocytes compared to wildtype males under calcium deficiency. This suggests that irisin reduces or prevents osteocytic osteolysis in males but not females.

In this same study^21^, while performing transcriptome analysis, we found that osteocytes from WT females had greater expression of genes associated with osteocytic osteolysis and osteoclastic bone resorption compared to WT males, which had greater expression of genes associated with steroid and fatty acid metabolism. Few differences were observed between female KO and WT osteocytes, but with calcium deficiency, genes responsible for osteocytic osteolysis and osteoclastic resorption were lower in the KO females than the WT females. This suggests that irisin ensures the survival of offspring to provide calcium for lactating females. Male KO mice have more but weaker bone compared to WT males, and when challenged with a low-calcium diet lost more bone than WT males. Male KO osteocytes had lower expression of genes associated with steroid and fatty acid metabolism, but higher expression of genes associated with bone resorption compared to male WT. This suggests that irisin plays a critical role in the development of the male but not the female skeleton and protects male but not female bone from calcium deficiency.

Our group has also conducted similar studies using 18–22-month-old wildtype and FNDC5 null male and female mice to examine the role of irisin in aging.^102^ We found that knocking out FNDC5/irisin did not affect bone phenotype under normal conditions. Aged female null mice continue to be partially protected against calcium-deficiency-induced bone loss, and the amount of bone loss is not age-dependent. On the other hand, male null mice continue to have higher bone mass until challenged. Under calcium-deficient conditions, aged male null mice lose more bone than their wildtype counterparts, and percentage of bone loss increases with age in these mice. This suggests that irisin is necessary to maintain and protect male bones especially with aging (Fig 3).

**Fig. 4:**
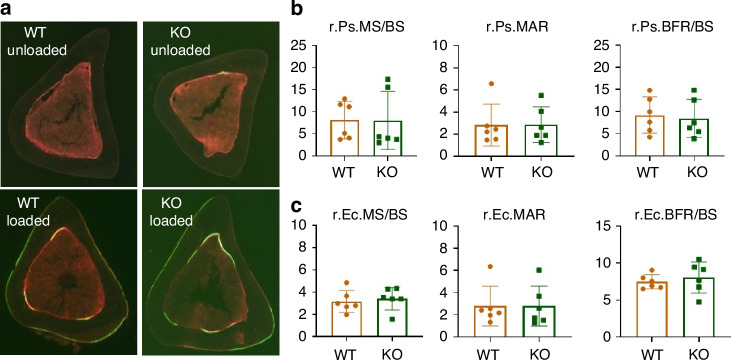
No direct influence of Fndc5/irisin deletion on bone anabolic response to loading (unpublished data). We performed anabolic loading and found no significant difference between male wildtype and mice lacking Fndc5/irisin (unpublished data). 5-month-old wildtype (WT) and FNDC5/irisin-null (KO) male mice were subjected to tibial axial loading under anesthesia for 220 cycles using a sinusoidal waveform of 2 Hz and peak force of 9 N 3X a week for 2 weeks as described previously^103^. Bones were labeled with calcein at the beginning of experiment and with alizarin red after the last loading session. Contralateral tibiae were used as controls (unloaded). Both groups had comparable mineral apposition rate and bone formation rate in both periosteal and endosteal bone. **a** representative images of unloaded and loaded tibia from WT and KO male mice, **b** periosteal bone parameters, **c** endosteal bone parameters. MS/BS mineral surface/bone surface, MAR mineral apposition rate, BFR bone formation rate. *n* = 6/per group

We have also performed studies examining the effects of lack of FNDC5/irisin in the murine hindlimb loading model. Our initial studies in male mice show no effect of *fndc5* global deletion in response to tibial axial loading (See Fig. 4). These studies suggest that irisin does not play a role in bone formation due to anabolic loading. However, studies by Colianni et al. show that conditioned media from murine myoblasts that underwent wheel running had a positive effect on osteoblast functions, potentially due to increased irisin.^58^ This suggests that a murine model of gene deletion provides different information compared to a murine irisin-overexpression model. Alternately, this may be due to irisin not having a direct function on bone formation at the physiological level whereas overexpression or overabundance of irisin may modulate bone formation. We have previously shown that L-BAIBA acts synergistically with sub-optimal tibial loading to increase mineral apposition and bone formation rate.^103^ As wheel running provides a sub-optimal load on bones as opposed to optimal load, perhaps irisin is working synergistically with sub-optimal loading by the same mechanism, but is not playing a role in bone response to optimal anabolic loading.

A summary of the different studies investigating the role of irisin in bone parameters and functions in vivo is presented below in Table 1.

**Table 1:** Studies investigating irisin and bone parameters in murine models

Study Model	Sex	Major Outcome	Reference
Global FNDC5/irisin KO mice using EIIa-cre for germline deletion, calcium-deficiency model	Females and males	Adult female KO mice are partially protected against lactation and dietary calcium deficiency-induced bone loss due to lower osteocytic osteolysis. Adult male KO mice are less resistant to dietary calcium deficiency-induced bone loss due to higher osteocytic osteolysis.	^21^
4 weeks of intraperitoneal r-irisin injection in 2-month-old mice	Male	Injected mice had increased cortical bone, strength, and bone formation rate.	^65^
4 weeks of intraperitoneal r-irisin injection in 8-week-old mice and fracture healing model	Male	Injected mice had accelerated fracture healing, and increased bone volume and BMC.	^66^
12-weeks of r-irisin injection in 6-8-week-old CKD mice	Male	Prevented renal osteodystrophy and increased osteoblast markers.	^69^
Different doses of r-irisin injection in 2- and 15-month-old mice	Male	Modulated BMSC lineage commitment, increased osteogenic differentiation and decreased adipogenic differentiation.	^70^
8-week-old mice with HA-tagged N-terminal FNDC5 into the gastrocnemius muscle	Male	Osteoblast activity increased only in groups with both aerobic exercise and irisin overexpression.	^71^
1 r-irisin injection at the femoral fracture site of 14-16-week-old rat	Male	Accelerated fracture healing, increased bone volume and strength.	^72^
12-week-old mice with hindlimb unloading or sciatic neurectomy	Male	Muscle FNDC5 RNA levels were positively correlated to trabecular BMD.	^77^
2-month-old mice with 4 weeks of hindlimb suspension and r-irisin injection	Male	Unloaded mice with r-irisin did not lose cortical BMD and lost less trabecular BMD compared to vehicle-treated unloaded mice.	^78^
4 weeks of r-irisin injection in 18-month-old mice	Male	The irisin-treated group had an increased Bcl2/Bax ratio in cortical bone.	^99^
Anterior cruciate ligament transection for knee osteoarthritis and 4 weeks of r-irisin injection in 3-month-old mice	Male	The irisin-treated group maintained their cartilage structure, had a higher subchondral trabecular bone, and decreased osteocyte apoptosis.	^100^
Orchidectomy (orx) and 8 weeks of r-irisin injection in 20-week-old mice	Male	Orx reduced muscle FNDC5 RNA. R-irisin-treated orx-ed mice had higher trabecular BMD compared to the vehicle-treated group.	^151^
Global FNDC5/irisin KO mice using EIIa-cre for germline deletion, Control and ovariectomy model	Female	No difference in cortical bone architecture, 9-month-old ovx-ed KO mice lost less bone due to lower osteocytic osteolysis and osteoclast resorption.	^16^
FNDC5 muscle overexpression model using an *Mck* promoter	Female	Overexpression mice had lower trabecular bone volume fraction and increased osteoclastogenesis.	^53^
5 weeks of twice-weekly r-irisin injection in ovx-ed mice	Female	r-irisin treated ovx-ed mice had improved bone parameters, increased osteoblast activity, and decreased inflammatory cytokines.	^73^
5 weeks of r-irisin injection in ovx-ed mice	Female	r-irisin treated ovx-ed mice had less trabecular bone loss and increased strength.	^79^
4 weeks of r-irisin injection in ovx-ed albino rats	Female	OVX-irisin-treated rats had increased femur dry and ash weight, and calcium and phosphorus levels.	^80^
Global irisin KO mice using TALEN technology and a frameshift mutation	Sex not mentioned	24-week-old KO mice had lower bone mass and strength.	^52^
Osterix-Cre FNDC5 KO mice (FNDC5/ irisin deleted in osteoblasts), wheel running exercise	Sex not mentioned	KO mice had delayed bone development and mineralization as well as decreased bone density. KO mice did not have any increase in bone volume fraction after wheel-running exercise.	^54^

### Sex differences are also observed in Alzheimer’s disease with regard to irisin

In addition to the potential sex differences regarding the role of irisin in bone, there also appear to be sex differences in the role of irisin in Alzheimer’s disease (AD). There are numerous differences when it comes to men and women with dementia and AD. Examples include the following: 1). Two-thirds of patients are female, 2). Rates of cognitive and functional decline are faster in women, 3) women with mild cognitive decline are more likely to progress to AD than men, and women show more global pathology than men.^104^ Correlations between irisin and dementia scores are stronger in women than men. Irisin levels in cerebrospinal fluid was found to be lower in both healthy and AD women compared to men.^105^ Irisin negatively correlates with amyloid positivity and fitness only in females.^106^ It was suggested that the relationship between physical activity and amyloid deposition is sex-specific and could be mediated by irisin. In mice transgenic for human tau, irisin reduced tau hyperphosphorylation in the hippocampus of female but not male mice.^107^

Irisin may not only have sex-specific effects on bone and cognition but also on other conditions. For example, irisin treatment decreased systemic and CNS inflammation in female mice, but exacerbated neuroinflammation in males.^107^ Another example is that hypertension and hypercholesterolemia in diabetic women is associated irisin polymorphism but not in men.^108^ These studies emphasize the importance of performing research, whether clinical or translational, on both males and females.

### Irisin expression and function in non-murine animal models

A recent study using domestic animals (pigs, ducks, horses) found FNDC5 mRNA and protein in the skeletal muscle and detected FNDC5/irisin in the serum by ELISA.^109^ Another study using chickens found FNDC5 mRNA expression in multiple tissues, including muscle and brain. FNDC5 mRNA expression increased in muscle during fasting and cold exposure.^110^ Fain et al. used castrated Rapacz FHM pigs (with a defect in their low-density lipoprotein receptor, resulting in hypercholesterolemia and atherosclerosis of the coronary vessels) and adult male Yucatan miniature swine (normal) on 16–20 week exercise regimen to find that FNDC5 mRNA and protein level remained unchanged in deltoid and triceps brachii muscles regardless of exercise. However, exercise increased plasma irisin only in the FHM and not the control pigs. The authors hypothesized this may be due to pigs having a non-functional UCP1.^111^ Basini et al. collected swine adipose stromal cells from abdominal subcutaneous adipose tissue and cultured them with or without irisin treatment. Irisin treatment decreased new DNA synthesis, a measure of cell proliferation, however increased ATP production, an indicator of metabolic activity.^112^ This result was similar to a previously published study from the same group using swine granulosa cells.^112^ A separate study by Hei et al. found that overexpression of FNDC5 via lentivirus increased preadipocyte differentiation in Mashen pigs, with no effect on ERK1/2 pathway.^113^ Interestingly, a study by Dong et al. using goats found that FNDC5 increases both proliferation and differentiation of goat adipose-derived stem cells.^114^ To date, there is no published data yet on the effect of irisin on non-murine, non-human bones.

### Reasons for the perceived contradictory effects of irisin

The findings from these studies investigating the effects of irisin on bone appear to be in conflict for females but not necessarily for males. However, it is important to consider the potential influence of other factors that may contribute to these discrepancies, including the sex and age of the mice used, the doses and duration of treatment, and the underlying differences in bone microstructure and strength between males and females and between young and aged individuals.^115^

The adult skeleton shows sexual dimorphism. Females usually have a lower bone density as well as a younger onset and a faster rate of bone loss compared to males.^116^ Murine models also exhibit similar sexual dimorphism with aging.^117–119^ This leads to a sex-dependent difference in gene expression profiles.^119^ Bone fracture healing response is different in males and females in both mice and humans.^120,121^ These differences in bone characteristics between sexes may lead to different responses to proteins, hormones, or treatments, including irisin. *However, this sexual dimorphism is often overlooked in study designs*. A recent review focused on the reporting of sex in bone research and has found that most studies do not report the sex of the mice used. Additionally, there is a bias toward including only male mice in the majority of the studies, except studies that used a gonadectomy procedure, where female mice were more frequently utilized.^122^ Clearly, there are significant sex difference in bone especially in response to challenges. Therefore, it is clear and necessary to include both sexes in studies to properly understand the effect of irisin on bone cells.

The key questions regarding the effects of irisin on osteocytes are: (1) how irisin specifically regulates osteocytes, and (2) whether male and female osteocytes respond similarly or differently to irisin. The osteocyte transcriptome differs significantly between male and female mice beginning at 4 weeks of age.^123^ Female osteocytes have higher expression of genes particularly involved in bone resorption pathways, including osteoclast differentiation, pH regulation, cation homeostasis and transport, proton transport, bone remodeling, and others.^21,123^ Female osteocytes provide calcium during lactation and other calcium-demanding conditions through osteocytic osteolysis. Of note, the genes highly expressed in female osteocytes compared to male osteocytes are the same ones necessary for osteocytic osteolysis to occur.^95,97^ These sets of data suggest that the sex difference of a divergent osteocyte transcriptome is most likely due to the need for calcium in milk for the successful survival of offspring. Female osteocytes are more primed than male osteocytes for bone resorption pathways to compensate for the increased calcium demand during reproduction. These findings highlight the importance of considering sex-specific differences in the study of bone health and the potential mechanisms by which irisin may affect osteocytes.

In our study focusing primarily on osteocytes, we observed sex differences in irisin’s effect on osteocytes both under normal conditions and during periods of calcium deficiency.^21^ Like the Youlten study,^123^ we observed a higher expression of osteocytic osteolysis genes in adult female osteocyte transcriptome compared to male osteocytes, thus they were primed for “resorption”. However, female and male FNDC5 null mice had no significant difference in these genes know to play a role in osteoclast resorption. This suggests a role of irisin modulating female osteocyte “priming” for resorption and calcium release. Additionally, we observed a lower expression of lipid and solute carrier genes and fatty acid metabolism genes in wildtype female osteocytes compared to wildtype male osteocytes, suggesting sex-dimorphic bioenergetics and metabolism. Interestingly, these differences are not significant between FNDC5-null female and male osteocytes. This indicates a sex-dependent role of irisin in modulating osteocyte bioenergetics. The sex difference prevails under conditions of calcium deficiency. Our study found that adult female mice lacking FNDC5 were partially protected from bone loss under conditions of increased calcium demand, while in contrast, adult male mice lacking FNDC5 were more vulnerable to bone loss.^21^ This suggests that irisin may have a beneficial effect on male osteocytes but may increase osteocytic osteolysis in female osteocytes. These findings help to reconcile many of the seemingly conflicting results from previous studies and highlight the importance of considering the role of sex in determining the function of irisin.

Aging also has a more severe effect on female bone microstructure and strength compared to males.^124^ A systematic review of body composition has demonstrated that older men have higher skeletal muscle mass compared to women, which is associated with better physical performance and bone density.^125^ In our study with aged mice, we observed both a sex and an age dependent effect of irisin on osteocytes.^21,102^ Therefore, the apparent duality in the effects of irisin on bone cells observed in these studies may be due, at least in part, to the sexual dimorphism of bones and the use of mice at different stages of development.

In many studies, there are discrepancies in how irisin has been administered, which may contribute to some of the conflicting results observed. Some studies have employed the continuous presence of irisin, while others have used an intermittent delivery method. The duration of elevated protein can significantly influence its effects on bone cells. Parathyroid hormone (PTH), a calcium homeostasis-regulating hormone,^126^ is the prime example of the opposing effects of continuous versus intermittent treatment. If PTH circulatory levels are chronically high, as in hyperparathyroidism, it induces bone resorption.^127^ Conversely, intermittent doses of PTH, for example, by daily injection, can have anabolic effects on bone and stimulate bone formation.^128^ Irisin may exhibit a similar timing effect. Genetically modified mouse models used in the study of FNDC5/irisin have lacked or overexpressed irisin, while studies using r-irisin have been administered on an intermittent basis. Additionally, different doses of irisin have been utilized in different studies, which may also be a factor regarding the observed discrepancies in its effects on bone cells. Our study used a genetic knockout model, in which irisin was continuously absent throughout the life of mice, which may provide insights into the mechanism of action of irisin especially in development.^21^

Irisin’s effect on bone may also vary based on physiological versus pathological conditions. Many in vitro studies have been conducted using cells cultured under physiological conditions, which may not accurately reflect the response of cells to challenge or stress. In vivo studies have been conducted under a range of conditions, including mechanical stimulation of bone and bone-muscle interactions in microgravity, unloading, fracture healing, and calcium deficiency studies. On the other hand, the absence of estrogen in ovariectomized mice has a different mechanism of action to induce bone loss compared to unloading.^129^ Estrogen, a sex hormone with a significant impact on bone health, is known to regulate bone remodeling. It is possible that irisin may regulate the functions of osteocyte mechanotransduction and perilacunar remodeling differently under different physiological and pathological conditions and hormone status such as ovariectomy. Further research is needed to clarify the mechanisms by which irisin may affect bone under various conditions.

So far, it is not known whether FNDC5, as an intact membrane protein, can act independently of its cleaved product.^130^ Genetic mouse models either deleting or overexpressing irisin inadvertently may change any potential function of FNDC5 as well. In contrast, treatment with r-irisin has been shown to increase irisin levels in serum, with no clear effect on FNDC5 mRNA levels. This raises the possibility that FNDC5 may have a distinct function from irisin on bone cells, which may help to explain some of the conflicting results observed in different studies to determine the impact of irisin on bone cells. Additionally, it is currently unknown whether FNDC5 has an autocrine effect on muscle that could influence the secretion of other myokines or metabolites and potentially affect osteocytes indirectly. Further focused research is necessary to understand the specific mechanisms by which FNDC5 and irisin may affect bone cells and to identify any potential differences in their functions.

### Human studies

To date, numerous human studies have been conducted to investigate the role of irisin in bone health under both physiological and pathological conditions. These studies have primarily utilized serum irisin measurements and correlated them with various bone parameters.

Studies conducted with children and young adults generally show a positive correlation between irisin and bone mass. In healthy young Caucasian individuals, irisin levels were higher in females after adjusting for lean mass.^131^ Multiple studies have found a positive correlation between serum irisin levels and bone mineral status in young individuals.^132–134^ One study observed a stronger correlation between serum irisin and BMD in females compared to males,^133^ whereas another study found that irisin had a positive correlation with sclerostin in males but a tendency towards a negative correlation in females. One study involving children with type 1 diabetes (T1DM) found significantly higher irisin levels in the T1DM group, which also positively correlated with CTX, PTH, and OCN. The researchers suggested a beneficial association between irisin and continuous insulin infusion for improving bone health outcomes in children with T1DM.^135^ Taken together, these studies suggest that irisin correlates positively with bone mass, with females having a stronger correlation and higher beneficial effects of irisin on bones.

A study with young adult females and males found that females had significantly lower serum sclerostin, DKK-1, irisin, and vitamin D levels. There was no correlation between irisin levels and muscle performance, however bone-specific physical activity was a significant predictor of irisin level.^136^ Another study found that young lean males with bed rest and decreased whole-body insulin sensitivity had significantly decreased irisin, young males on bed rest with increased whole-body insulin sensitivity had no difference in irisin levels. Bone loss was not measured in this study.^137^ Another study on pediatric cancer survivors found no correlation between irisin and bone turnover markers.^138^ These findings suggest that irisin may have complex relationships with various markers of bone health in young adults and that these relationships may differ between males and females.

Aging is usually accompanied by a decrease in physical activity, which is associated with chronic diseases. One of the highly recommended means to delay the onset of aging and associated diseases is exercise.^139,140^ Irisin level decreases with age, which can be partially recovered with exercise.^141,142^ The decrease in irisin levels may be due to the decrease in muscle mass, the primary producer of irisin, with aging, or the overall decrease in activity level leading to a decrease in total muscle contraction. The mechanism responsible for this decrease is not yet known. In a study comparing young and middle-aged to older individuals, young people had higher serum irisin compared to middle-aged people (male and female). However, with 8 weeks of endurance training, irisin level significantly increased in middle-aged to older people but not in young subjects, suggesting an age-specific effect of exercise on irisin levels.^143^ A study of Chinese Han people aged 65 years and older found significantly higher irisin levels in females compared to males, in contrast to the study described above,^136^ with a significantly higher irisin level in males with higher BMD. This association was not observed in females.^144^ The observed sexual dimorphism in irisin levels in older individuals is consistent with the findings of the previously mentioned study in younger individuals.^131^ The discrepancy in irisin levels between females and males may be due to changes with aging, different population, or differences in measurement levels. Of note, serum irisin levels measured in these two studies are 5–7× time different from each other.

Aging is frequently accompanied by bone loss in the form of osteopenia and osteoporosis, as well as muscle loss known as sarcopenia. These two age-related conditions often occur together, leading to a decline in bone and muscle mass and strength.^45^ Irisin is positively associated with BMD in older individuals, with lower circulating irisin levels in osteopenic and osteoporotic individuals.^142,145^ A meta-analysis concluded that irisin was positively correlated with BMD, and the circulating level decreased in osteoporotic patients. Two of these studies included both females and males, and five included postmenopausal women.^146^ Several studies have investigated the association between irisin levels and fragility fracture, and most have found a negative correlation between irisin and prevalent fracture.^130,147^ Of note, different methods and commercially available kits were used to determine irisin levels in these studies, which may explain the large differences in serum levels. Taken together, decrease of irisin is correlated to age-related bone diseases, however more studies need to be carried out to understand the causal vs correlation effect of irisin on bone, as well as sex dependency with increasing age.

A summary of different human studies looking at the association between circulating irisin levels and bone parameters is included in Table 2.

**Table 2:** Studies investigating irisin and bone parameters in humans

Studied Population	Studied sex	Measured Irisin Level	Major Outcome	Reference
Children (6–8 years) from Finland	Male and female	151.5 ng/mL	Irisin positively correlated with BMD	^133^
Healthy Caucasian children (9.82 ± 3.252 years)	Male and female	2.59 µg/mL (2 590 ng/mL)	Irisin positively correlated to bone mineral status and serum OCN, negatively correlated to DKK-1	^134^
Children and adolescents with T1DM (12.2 ± 4 years)	Male and female	T1DM: 3.43 µg/mL (3 430 ng/mL) Control: 2.57 µg/mL (2 570 ng/mL)	Patients with higher serum irisin had better bone health	^135^
Adolescent athletes (14–21 years)	Female	Amenorrheic athletes: 3.2 mcg/mL (3 200 ng/mL) Eumenorrheic athletes: 3.85 mcg/mL (3 850 ng/mL) Non-athletes: 3.6 mcg/mL (3 600 ng/mL)	Irisin positively correlated to BMD, stiffness, and failure load	^132^
Caucasian football (soccer) players (24.7 ± 1.2 years)	Male	3.5 µg/ml (3 500 ng/mL)	Irisin positively correlated to total and sub-regional BMD	^152^
Adults with functional hypothalamic amenorrhea (FHA) (18–34 years)	Female	FHA: 7.67 (unit not mentioned) Log irisin: 2.03 Control: 11.28(unit not mentioned) Log irisin: 2.42	Lower irisin level correlated to lower BMD	^153^
Becker muscular dystrophy patients (36 years median age)	Male	Controls: 12.90 ng/mL Patients: 12.15 ng/mL	Patients with lower BMD had higher levels of serum irisin	^154^
Young (21 ± 1 years) and middle-aged to older individuals (65 ± 8 years)	Male and female	Middle-aged: Control: 140.6 ng/mL Exercised: 168.7 ng/mL Young: Control: 155.3 ng/mL Exercised: 157 ng/mL	8 weeks of endurance training increased irisin level in middle-aged people	^143^
T2DM patients (51.08 ± 12.67 years) and controls (50.04 ± 8.98 years)	Male and female	Controls: 11.69 ± 2.06 ng/mL Patients: 10.90 ± 1.88 ng/mL	Irisin did not correlate to lumber BMD, and was negatively correlated to CTX	^155^
Postmenopausal Caucasian women (>65 years)	Female	Control: 50.4 ± 2.7 ng/mL Denosumab-treated patients: 53.0 ± 2.3 ng/mL Teriparatide-treated patients: 50.4 ± 3.5 ng/mL	Irisin negatively correlated to osteoporotic fracture but not to bone mass	^156^
Chinese Han (>65 years)	Male and female	Females: 27.28 ng/mL, Males: 17.16 ng/mL	Irisin positively correlated to BMD only in males	^144^
Geriatric Chinese men (60–75 years)	Male	Control: 422.13 ng/mL, Osteopenia: 184.37 ng/mL, Osteoporosis: 159.68 ng/mL	Irisin correlated to BMD, serum irisin level was lower in osteopenic and osteoporotic patients	^145^
Patients undergoing total hip or knee replacement (68.71 ± 12.31 years)	Male and female	Females: 3.656 mg/mL (3 656 ng/mL) Males: 2.980 mg/mL (2 980 ng/mL)	Irisin positively correlated to BMD, serum irisin level was lower in osteopenic and osteoporotic patients	^142^

In summary, irisin appears to have a positive association with bone mass and BMD in young individuals, especially in females. However, with age, this association becomes less clear. This may be due to changes in physical activity levels, lifestyle, changes in hormone levels, underlying genetic or disease conditions, and so on.

### Issues concerning the quantification of irisin

The quantification of irisin has been a subject of concern as outlined in recent scientific literature. A majority of the in vitro and in vivo animal studies and clinical studies have employed different commercially available enzyme-linked immunosorbent assay (ELISA) kits to measure irisin levels. However, these studies have reported significantly different irisin levels, ranging from 13.5 pg/mL to 14.8 µg/mL in humans and <1 pg/mL to >1.5 µg/mL in mice.^23^ Several publications have raised questions about the validity of these measurements.^23,130,148,149^ Possible reasons for this discrepancy include the lack of specificity of the antibodies used as well as cross-reactivity with homologous proteins. Issues have arisen with Western blotting to measure irisin as well. Several commercially available antibodies target the C-terminal region of FNDC5, which is absent from the cleaved irisin. As such, these antibodies should not be able to detect irisin. The commercially available antibodies supposedly specific to irisin appear to also have cross-reactivity and non-specific binding. Furthermore, the post-translational modification of glycosylation of irisin dimer necessitates deglycosylation prior to the western blotting, as differences in glycosylation can result in bands of different sizes. Additionally, FNDC5/irisin shares sequence homology with the other fibronection domain-containing proteins. All of these have made measuring irisin in a reproducible manner challenging. Mass spectrometry, an antibody-independent method of measuring proteins, has been employed to quantify irisin levels. Jedrichowsky et al. have measured 3.6 ng/mL irisin in sedentary individuals, which increased to approximately 4.3 ng/mL in exercised individuals.^150^ In mice, the reported irisin level was 0.3 ng/mL,^16^ whereas, in rats, it was 0.6–0.9 ng/mL^23^ via quantitative mass spectrometry. These levels are significantly lower than those reported with techniques such as western blotting or ELISAs. Further testing and validation of the methodology for irisin quantitation are necessary to establish a more accurate and reliable method to accurately quantify irisin.

### Concluding remarks

Recent years have seen a surge in research focused on the role of myokines and their effects on numerous tissues, both locally and distant. Among the myokines, irisin has emerged as one of the leading candidates for investigation. A growing body of evidence supports the paracrine function of irisin in tissues such as adipose, brain, and liver, among others. In particular, the bone-muscle interaction has garnered significant attention, and the effects of irisin on bone cells, including osteoblasts, osteoclasts, and osteocytes, have been the subject of intensive investigation. In this review, we summarized the effects of irisin on bone cells as reported in the literature, highlighting differences across in vitro, ex vivo, in vivo study models and clinical studies. We address the limitations and challenges associated with current research, including the need for improved study models and more reliable quantification methods.

The amount of data showing positive effects of irisin on the brain, fat, heart, bone and other tissues is impressive; several questions remain. Key questions regarding the mechanism of action and functional significance of irisin in bone cells remain unanswered. Different studies have demonstrated that irisin promotes the proliferation and differentiation of osteoblasts both in vitro and in vivo. However, the findings regarding its effect on osteoclast function have been inconsistent, with some studies finding inhibition and others stimulatory effects. Some studies have proposed that irisin impacts osteoclast precursor proliferation and differentiation, although the direction and mechanisms of this impact remain to be determined. The role of irisin in regulating osteocyte functions is also controversial. The majority of studies show that irisin is an osteocyte viability factor. However, its role in osteocytic osteolysis remains unclear. There is a strong indication of sex and age-based differences in irisin’s effect on osteocytes. Sex differences in the role of irisin in dementia and Alzheimer’s disease has been described. There are likely other effects that have been missed due to a greater disposition and focus of investigators on the use of male mice. Further studies are required to fully understand the underlying mechanisms involved and clarify the function of irisin in both male and female bone.

Accurate quantification of irisin levels remains a challenge in the field, and the development of reliable and reproducible assay methods is critical. Anti-irisin antibodies have been shown to be non-specific and some assays have been described as non-specific. Until an assay is available that is clearly specific for irisin, studies performed using these antibodies and kits must take this into consideration when interpreting results.

There are several potential explanations for the conflicting results in studies examining the effects of irisin on bone. As emphasized in this review, sex should be addressed with both males and females included in studies. Second, the dose and timing of delivery of irisin could also be a factor. It is well known that drugs can have very different effects on men compared to women and that dose should also be adjusted for sex. The third is whether irisin is being administered intermittently or continuously. History has taught us that intermittent doses of factors such as parathyroid hormone, interleukin 6, and others have positive effects while continuous elevated levels have serious negative effects. Fourth, models of gene deletion or gene overexpression yield very useful information, however these models yield information distinct from delivery models. Information from these studies will have significant implications for the treatment and prevention of bone-related disorders, such as osteoporosis, osteoarthritis, aging, calcium deficiency-related bone loss, and the overall maintenance of bone health.

There has been a tendency in the literature to attribute all of the positive effects of exercise to irisin. Irisin is only one of many myokines and metabolites produced by contracted muscle. It is highly likely that these muscle-secreted factors work synergistically along with other circulating factors induced by exercise such as osteokines. Future studies should test to determine if the addition or combination of irisin with other exerkines is additive or synergistic. Resistance exercise builds bone better than aerobic exercise. It would be important to determine the magnitude and duration of irisin production in different forms of exercise, alone and in combination. These questions highlight the need for comprehensive studies to resolve outstanding questions to advance our understanding of the role of irisin in bone-muscle interaction.
